# Case Report: Perivascular epithelioid tumors of the gastrointestinal tract

**DOI:** 10.3389/fonc.2022.1026825

**Published:** 2023-01-16

**Authors:** Hui Yan, Shuhui Zhang, Ying Ba, Kun Li, Guoling Gao, Yanmin Li, Yan Zhang, Chengxia Liu, Ning Shi

**Affiliations:** ^1^ Department of Gastroenterology, Binzhou Medical University Hospital, Binzhou, Shandong, China; ^2^ Department of Pathology, Binzhou Medical University Hospital, Binzhou, Shandong, China

**Keywords:** perivascular epithelioid cell tumors, sigmoid colon, HMB-45, treatment, case report

## Abstract

**Background:**

Perivascular epithelioid cell tumor of the gastrointestinal tract (GI PEComa) is a rare mesenchymal neoplasm. GI PEComa is mostly observed in the colon and has a marked middle-aged female predominance. PEComa has no typical clinical or imaging manifestations or endoscopic characteristics. Therefore, the diagnosis of this disease mostly relies on pathological findings. HMB-45 is a sensitive immune marker of PEComa.

**Case presentation:**

We reported a case of a middle-aged female with sigmoid colon PEComa. To exclude carcinogenesis, the large basal polyp in the sigmoid colon was removed by endoscopic mucosal resection (EMR). Immunohistochemistry analysis results showed that this lesion expressed HMB-45, which is a characteristic melanin marker of PEComa. Finally, the lesion was diagnosed as sigmoid colon PEComa. At the time of submission of this report, surgical resection was the primary treatment for PEComa. Though the characteristics of tumor biology and clinical behavior in PEComa are not clear, the boundary is clear, and the tumor can be completely removed. However, close follow-up is required after the surgery because of the lesion’s undetermined benign and malignant nature.

**Conclusion:**

The present case study emphasizes the importance of pathological diagnosis. Therefore, upon finding gastrointestinal polyps with a mucosal ulcer under endoscopy, the GI PEComa diagnosis should be considered. It is necessary to detect the characteristic melanin markers of PEComa. Due to the rarity of these cases, challenges are faced in diagnosing and treating PEComa.

## Introduction

**Figure 1:**
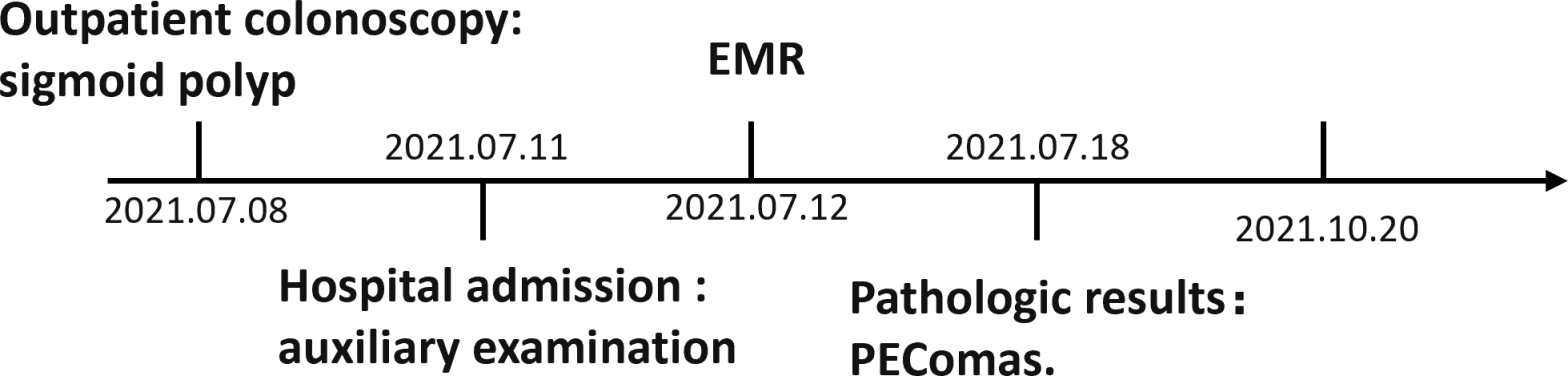
The case history.

PEComa is a family of rare mesenchymal neoplasms with a marked female predominance. This disease’s peak onset is observed in 40-49 years of age. In addition, a few cases occur in children or adolescents (1). In 1992, Bonetti et al. used “perivascular epithelioid cells (PEC)” to describe certain epithelioid cells with perivascular distribution and expression of melanocyte markers (2). In 2002, the WHO defined PEComa as “a mesenchymal tumor composed of histologically and immunohistochemically distinctive perivascular epithelioid cells” (3). The PEComa family mainly includes angiomyolipoma (AML), lymphangioleiomyomatosis (LAM), clear-cell “sugar” tumor (CCST) of the lung, and malignant ligament clear cell tumor (CCMT) (4, 5). In addition, a rare group of PEComas called “PEComas–not otherwise specified” (PEComas-NOS), with similar morphology and immunophenotype, may arise in soft tissues (such as retroperitoneal, abdominopelvic, and cutaneous) and visceral sites (such as gastrointestinal, gynecologic, and genitourinary) (6, 7). In immunohistochemistry, virtually all PEComas express melanocytic markers, such as HMB-45 and Melan-A (5, 8, 9). PEComas exhibit neither characteristic clinical nor typical imaging and endoscopic manifestations. Therefore, PEComas are generally diagnosed using typical histological and immunohistochemical findings. PEComas-NOS manifests a broad biological behavior including benign, uncertain malignant potential, and malignant features. Folpe et al. proposed that “tumor size >5 cm, infiltrative growth pattern, high nuclear grade, necrosis, and mitotic activity >1/50 HPF” could predict malignant behavior of PEComas (10) Moreover, these features are the key to the prognostication of PEComa. Currently, wide-margin surgical resection is the best treatment method for PEComas. Additionally, some targeted molecular therapies are under the exploratory phase. Here, we present a case of PEComa of gastrointestinal tract located in the sigmoid colon of a female patient. The clinical and endoscopic findings and pathological features of this case are described to deepen our understanding of GI PEComa so as to improve its diagnostic accuracy and therapeutic effect (**Figure 1**).

## Case presentation

A 47-year-old female was admitted to Binzhou Medical university hospital. The patient had no tumor-related family history or medical history of tuberous sclerosis complex (TSC), inflammatory bowel diseases, or malignant melanoma. Her physical examination results were within normal ranges. The blood and biochemical parameters, including the tumor markers such as the levels of serum CEA, CA19-9, AFP, CA15-3, and CA-125, were also within the normal ranges. The patient underwent a colonoscopy at the outpatient department of our hospital for constipation. The examination revealed a 1.5 cm × 2.0 cm polyp with surface erosion in the colon, 18 cm away from the anus. The polyp had a large basal lesion with an eroded surface. The patient was referred to our inpatient department for polyp resection. Although we considered the possibility of neoplastic polyps, we did not consider the possibility of GI PEComa initially.

**Figure 2:**
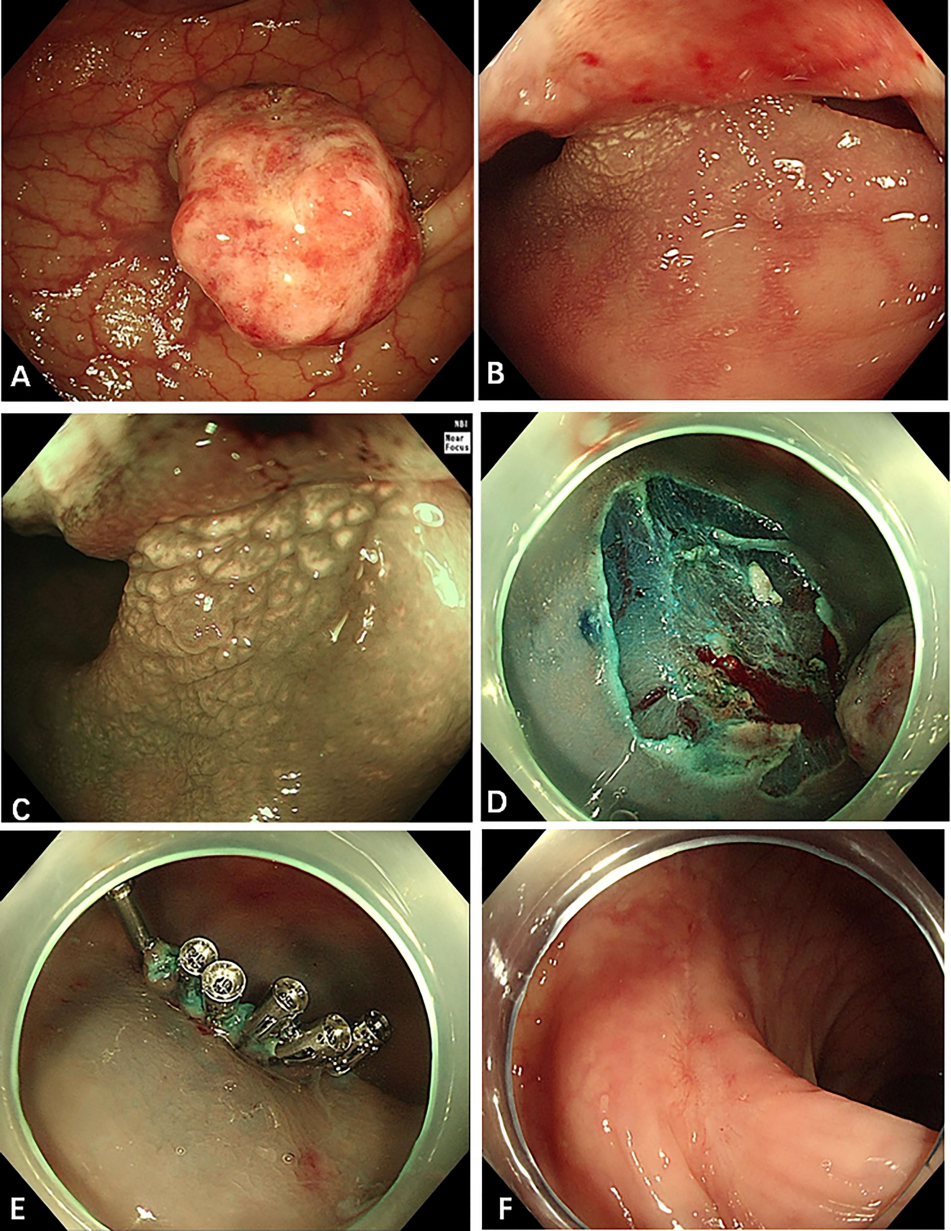
Endoscopic appearance of the sigmoid colon tumor. **(A–C)** There was a polypoid tumor in the sigmoid colon, 18 cm away from the anus, with a diameter of 1.5 cm × 2 cm and with a wide base. The surface of the polyp is eroded, and the gland tube and blood vessel under (Narrow Band Imaging, NBI) are not clearly observed. But the gland tube at the base is still regular. **(D, E)** A little blood oozed from the wound after EMR. We applied six endoscopic hemoclips to stop the bleeding. **(F)** Three months after the EMR, the wound was observed to be healed well.

The well-circumscribed tumor was 2.0 cm in maximum diameter and had a wide base (**Figures 2A–C**). Because the tumor had a wide base, we used endoscopic mucosal resection (EMR) to remove the lesion (**Figures 2D, E**) and improve postoperative pathology.

**Figure 3:**
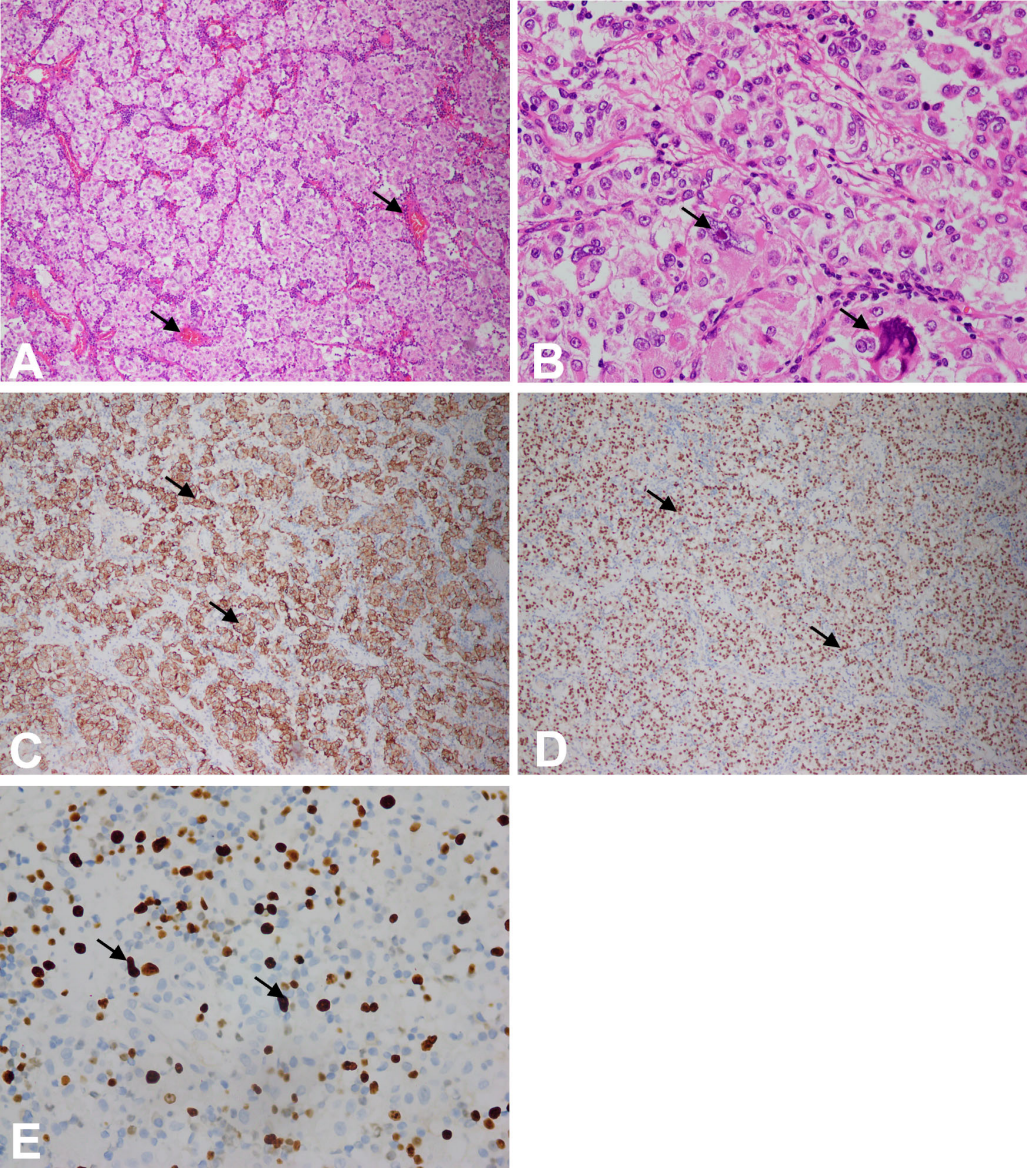
**(A)** Microscopic features of the tumor at 10× magnification using microscope. The tumor consisted of nests of epithelioid cells with clear-to-eosinophilic granular cytoplasm, as pointed by arrowheads. **(B)** Microscopic features of the tumor at 40× magnification using microscope. The tumor cells had large nuclei with prominent nucleoli, and the local atypia of tumor cells was obvious, as pointed by arrowheads. **(C)** The tumor cells were immunoreactive for HMB-45, and yellow was positive in the cytoplasm, as pointed by arrowheads (at 10× magnification using microscope). **(D)** The tumor cells were immunoreactive for TFE-3, and yellow was positive in the nucleus, as pointed by arrowheads (at 10x magnification using microscope). **(E)** Ki67 immunohistochemistry at 40× magnification using microscope.

Microscopically, the tumor is composed of nests of round, oval, or polygonal epithelioid cells with abundant clear eosinophilic granular cytoplasm. In our case, the nests were separated by thin fibrovascular septa (**Figure 3A**). The tumor cells had large nuclei with prominent nucleoli and the obvious local atypia of tumor cells (**Figure 3B**). The mitotic rate was >1/50HPF and about 20% of the cells expressed Ki67 (**Figure 3E**). In addition, the tumor showed invasive growth and foci of coagulation necrosis.

**Figure 4:**
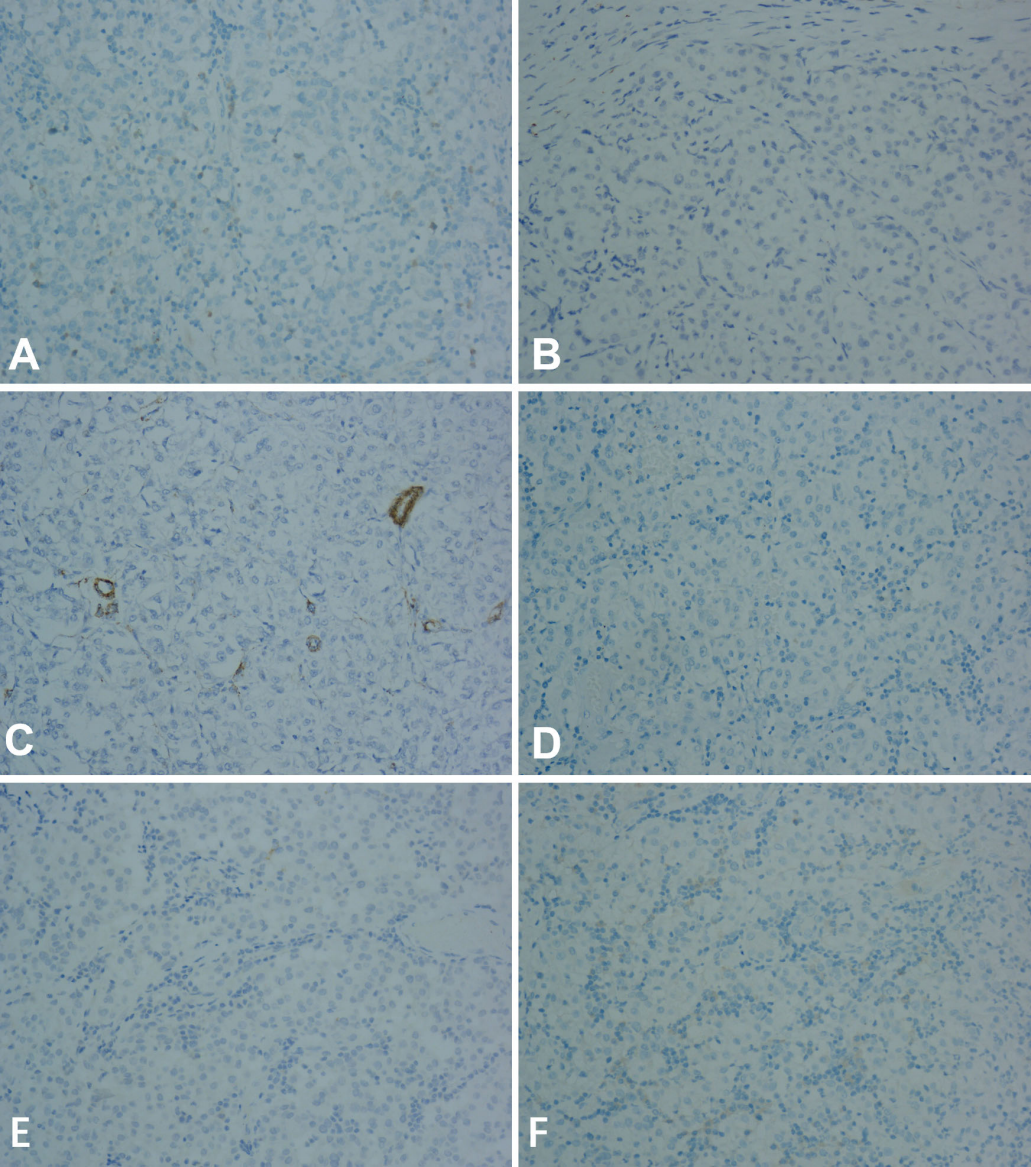
The tumor was negative for Melan-A, desmin, smooth muscle actin (SMA), S-100, CD117, and CK at 20x magnification using microscope. **(A)** Melan-A. **(B)** desmin. **(C)** smooth muscle actin (SMA). **(D)** S-100. **(E)** CK. **(F)** CD117.

Immunohistochemically, the cells stained positive for HMB-45 (**Figure 3C**) and TFE-3 (**Figure 3D**). However, they were negative for Melan-A, desmin, smooth muscle actin (SMA), S-100, CD117, and CK (**Figure 4**). Therefore, depending on the endoscopy, histology, and immunohistochemistry results, we diagnosed the tumor as GI PEComa. Of note, we did not find any vascular invasion and base incision margin involvement in the postoperative pathology, which showed complete curative resection.

Given the tumor invasive growth, obvious local atypia, focal coagulation necrosis, mitotic rate >1/50HPF, and the high expression of Ki67, the possibility of malignancy was high. Therefore, we used chest X-ray, total abdominal CT, and gastroscopy to exclude metastasis.

We also conducted relevant laboratory and imaging examinations. The examinations did not show any signs of malignancy or metastasis. Combined with pathological findings, the case was diagnosed as GI PEComa with unclear benign and malignant origin. Therefore, a long-term follow-up was necessary to exclude tumor malignant transformation, recurrence, and metastasis. Three months after EMR, colonoscopy showed that the postoperative healing was good (**Figure 2F**). The laboratory examinations and abdominal and pelvic CT showed no signs of recurrence and metastasis.

## Discussion

The gastrointestinal tract is the second most common location, after the uterus, of PEComa, accounting for 20-25% of the cases. The colon is the most common site of GI PEComa, followed by the small intestine, rectum, and stomach (11). Although PEComa shows a significant female predominance, the frequency of GI PEComa is similar in female and male patients (12). Based on previous case reports, the biological behavior of GI PEComa varies from benign to malignant. Most GI PEComas are benign or have uncertain malignant potential. However, a few cases show malignant behavior. Compared to other body parts, the malignancy rate of GI PEComa is relatively high (10–15).

Upon endoscopy examination, most GI PEComas exhibit clear edges, mostly polypoid lesions, necrosis, and mucosal ulcer. In previous reports, some GI PEComas were limited to the mucosa and submucosa, however, some extended to the muscular propria or even into the mesentery (12). Therefore, we could not diagnose GI PEComa only by the general manifestations observed during endoscopy. The final diagnosis depended on the pathological findings. Histologically, PEComas are composed of nests of epithelioid cells, have abundant granular eosinophilic to clear cytoplasm, and are surrounded by a delicate capillary vasculature (16). In most cases, the tumor shows a nested, trabecular, or sheet-like architecture. In addition, the tumors often show prominent nuclear pleomorphism, including coarse chromatin, hyperchromasia, prominent nucleoli, and pleomorphism (12). Immunohistochemically, PEComas express both melanocyte and muscle markers (5). Of note, the most sensitive markers of melanocytes are HMB-45 and Melan-A (17). In our case, the tumor was described as a polyp or polypoid, was well-circumscribed, and had no specific diagnostic value. Initially, we did not consider the possibility of GI PEComa. However, sigmoid colon PEComa was diagnosed after the pathological biopsy due to its positive characteristic immunohistochemical features. PEComa may have TSC mutation and TFE3 gene fusion. However, no cytogenetic and genomic analyses of the tumor were performed.

Differential diagnosis with other gastrointestinal tumors is important. A benign submucosal tumor and gastrointestinal stromal tumor (GIST) have similar endoscopic and histological appearances as GI PEComa. However, the melanin (HBM45 and Melan-A) expression is typically negative in these tumors but always positive in PEComas. In addition, PEComas must be distinguished from melanoma, liomyoma, etc based on the results of the immunohistochemical analysis. Our case showed that the possibility of GI PEComa could not be ignored for polypoid lesions. Therefore, it was essential to perform histological and immunohistochemical examinations.

Most GI PEComas are benign or have uncertain malignant potential and do not metastasize. However, malignant PEComas demonstrate local invasion and/or metastatic spread. The optimal approach to treat PEComa is not yet clear (18). National Comprehensive Cancer Network (NCCN) guidelines indicate that surgical resection is the mainstay of treatment of GI PEComas, particularly for benign tumors (19). Moreover, in the current guidelines, no standardized regimen is provided to avoid its recurrence after surgery. In the current case report, malignant PEComa with metastasis was treated with adjuvant chemotherapy (20–23); however, a standard chemotherapeutic regimen is not established for advanced PEComa.

Presently, the knowledge about the molecular genetic alterations in PEComas is limited. In a previous report, two different molecular groups were identified, including the classic marker of TSC mutation and *TFE3* gene fusion. Further, molecular genetic studies revealed the deletion of 16p at the locus of the *TSC2* gene in PEComas. *TSC1* and *TSC2* genes negatively regulated the activation of mTOR (24). Therefore, for the palliative therapy of PEComas, mTOR inhibitors, such as sirolimus and everolimus, can be used in patients with TSC mutations (25). Also, targeting the VEGF/VEGFR signaling pathway may play an important role in the inhibition of tumorigenesis. Furthermore, it may be a viable treatment option for TFE3-related malignant PEComas. Studies have shown that VEGFR-2 inhibitor apatinib has therapeutic potential in PEComa patients with TFE3 rearrangement (26). Therefore, targeting the VEGF/VEGFR signaling pathway may be a novel therapeutic option for TFE3-associated malignant PEComas. However, the clinical cases are limited. Thus, combination-targeted therapy needs further exploration. Recently, Maren Schmiester found that the TSC1/2-mTOR pathway and TFE3 overexpression can promote tumorigenesis of PEComa (27).

We used endoscopic mucosal resection (EMR) to remove the tumor. EMR is a new endoscopic minimally invasive treatment with the advantages of lesser trauma, complete resection of lesion mucosa, and fewer complications. Although EMR has several advantages, such as fewer procedural complications, its use for PEComas is not the first treatment recommendation. Moreover, EMR for PEComa is not validated, and prospective data of PEComa patients are lacking. By differentiating between benign and malignant PEComas, EMR can potentially undertreat more aggressive diseases. Nonetheless, we did not find signs of malignancy and metastasis in our case; thus, no additional surgery or other adjuvant treatment was required.

Due to the rarity and the lack of standardized biological manifestation of PEComas, the diagnostic criteria of malignant PEComas are not fully agreed upon internationally. Therefore, the prognosis of PEComas, too, remains uncertain (28–30). However, the malignant behavior may be predicted by histological features, such as size more than 5 cm, invasive growth, high differentiation, mitotic rate ≥1/50HPF, necrosis, and vascular infiltration (13). Though the tumor size was less than 5 cm in the present case, coagulative necrosis was observed, showing that the PEComa had potential invasive growth with an uncertainty of being malignant. Therefore, a long-term follow-up with the patient is essential, throughout which endoscopy and imaging examinations will be performed regularly to exclude tumor recurrence and distant metastasis. So far, the patient has no specific symptoms of discomfort. No recurrence or distant metastases were observed during follow-up of 3 mo.

## Conclusion

GI PEComas have no typical clinical and imaging manifestations or endoscopic characteristics. Thus, it is difficult to diagnose the tumor using these parameters and even ignore the possibility of PEComas. In the case of gastrointestinal polyps, especially mucosal ulcers, the possibility of GI PEComa should be considered. It is necessary to perform a pathological biopsy and immunohistochemical analysis of the excised tissues to assess the characteristic melanin markers of PEComas and confirm the benign or malignant nature of the lesion. Due to the rarity of GI PEComas and the limitation of clinical cases, we face challenges in diagnosing and treating them.

## Data availability statement

The original contributions presented in the study are included in the article/supplementary material. Further inquiries can be directed to the corresponding author.

## Ethics statement

Written informed consent was obtained from the individual(s) for the publication of any potentially identifiable images or data included in this article.

## Author contributions

HY: Primary author (wrote most of the paper). YB and KL: Author of the manuscript, gastroenterologist involved in clinical management. GG and YL: Provided grammatical corrections to the manuscript. YZ was the pathologists responsible for the pathological diagnosis. CL: Provided reviews to the scientific content of the manuscript. NS: provided revisions to the scientific content of the manuscript. All authors contributed to the article and approved the submitted version.
